# Medication Use in Multiple Sclerosis: A Population‐Based Comparison With the General Danish Population

**DOI:** 10.1002/acn3.70155

**Published:** 2025-07-31

**Authors:** Josefine Windfeld‐Mathiasen, Henrik Horwitz, Ida M. Heerfordt, Elisabeth Framke, Melinda Magyari

**Affiliations:** ^1^ Department of Clinical Pharmacology Copenhagen University Hospital ‐ Bispebjerg and Frederiksberg Copenhagen Denmark; ^2^ Department of Clinical Medicine University of Copenhagen Copenhagen Denmark; ^3^ Department of Neurology, The Danish Multiple Sclerosis Registry Copenhagen University Hospital – Rigshospitalet Glostrup Denmark; ^4^ Department of Geriatric and Palliative Medicine Copenhagen University Hospital ‐ Bispebjerg and Frederiksberg Copenhagen Denmark; ^5^ Danish Multiple Sclerosis Center, Department of Neurology Copenhagen University Hospital – Rigshospitalet Glostrup Denmark

**Keywords:** chronic disease management, drug utilization, neuroimmunology, pharmacoepidemiology, population‐based study, prescription patterns

## Abstract

**Objective:**

To investigate the overall use of prescription medications among individuals with multiple sclerosis compared to the general population, with a focus on treatments beyond disease‐modifying therapies.

**Methods:**

We conducted a nationwide, registry‐based study in Denmark. All residents with a diagnosis of multiple sclerosis aged 20–80 years and alive on January 1, 2023, were included and followed for 1 year. Each patient was matched with 10 individuals from the general population based on age, sex, and residence. Prescription data were obtained from the Danish National Prescription Registry and categorized using the Anatomical Therapeutic Chemical classification. Relative risks of medication use were calculated.

**Results:**

A total of 14,491 individuals with multiple sclerosis and 144,910 matched controls were included. Patients with multiple sclerosis had significantly higher use of several therapeutic drug classes, including analgesics (51% vs. 33%; RR: 1.56, 95% CI: 1.52–1.60), agents for constipation (12% vs. 3%; RR: 4.61, 95% CI: 4.36–4.88), spasmolytics, and urological medications. Among specific drugs, the most pronounced differences were observed for modafinil (RR: 323.33, 95% CI: 228.67–457.19), baclofen (RR: 36.51, 95% CI: 33.33–39.99), and fesoterodine (RR: 36.36, 95% CI: 18.66–70.87). In contrast, individuals with multiple sclerosis had lower usage of antidiabetic medications and treatments for chronic obstructive pulmonary disease.

**Interpretation:**

This large, population‐based study reveals extensive use of medications targeting symptoms and comorbidities in individuals with multiple sclerosis. These insights can guide healthcare providers in optimizing patient management, addressing overlooked treatment needs, and improving overall care.

## 1 Introduction

Multiple sclerosis (MS) is the most common neuroimmunological disease of the central nervous system [1, 2]. The prevalence of MS has been steadily increasing since 1950, and owing to its lifelong nature and the rising incidence, it has reached its highest prevalence level to date [3].

The symptoms of MS are highly varied, including fatigue, cognitive changes, paralysis, spasticity, and bladder dysfunction [1, 2]. Additionally, individuals with MS have a well‐documented increased risk of comorbidities such as depression, anxiety, cerebrovascular and cardiovascular diseases, and certain autoimmune disorders [4]. MS with somatic comorbidities is associated with lower income levels after disease onset [5], and these comorbidities have also been linked to diagnostic delays and excess mortality among MS patients [6]. However, there are few reports on the pharmacological treatment of MS symptoms and comorbidities [7, 8, 9, 10]. Understanding the frequency of medication use in MS can highlight areas needing attention in patient care and support more evidence‐based approaches to polypharmacy management in MS.

## 2 Aim

This study aimed to examine differences in prescription medication use between MS patients and a matched control group from the Danish population without MS.

## 3 Methods

### 3.1 Data Collection

**FIGURE 1:**
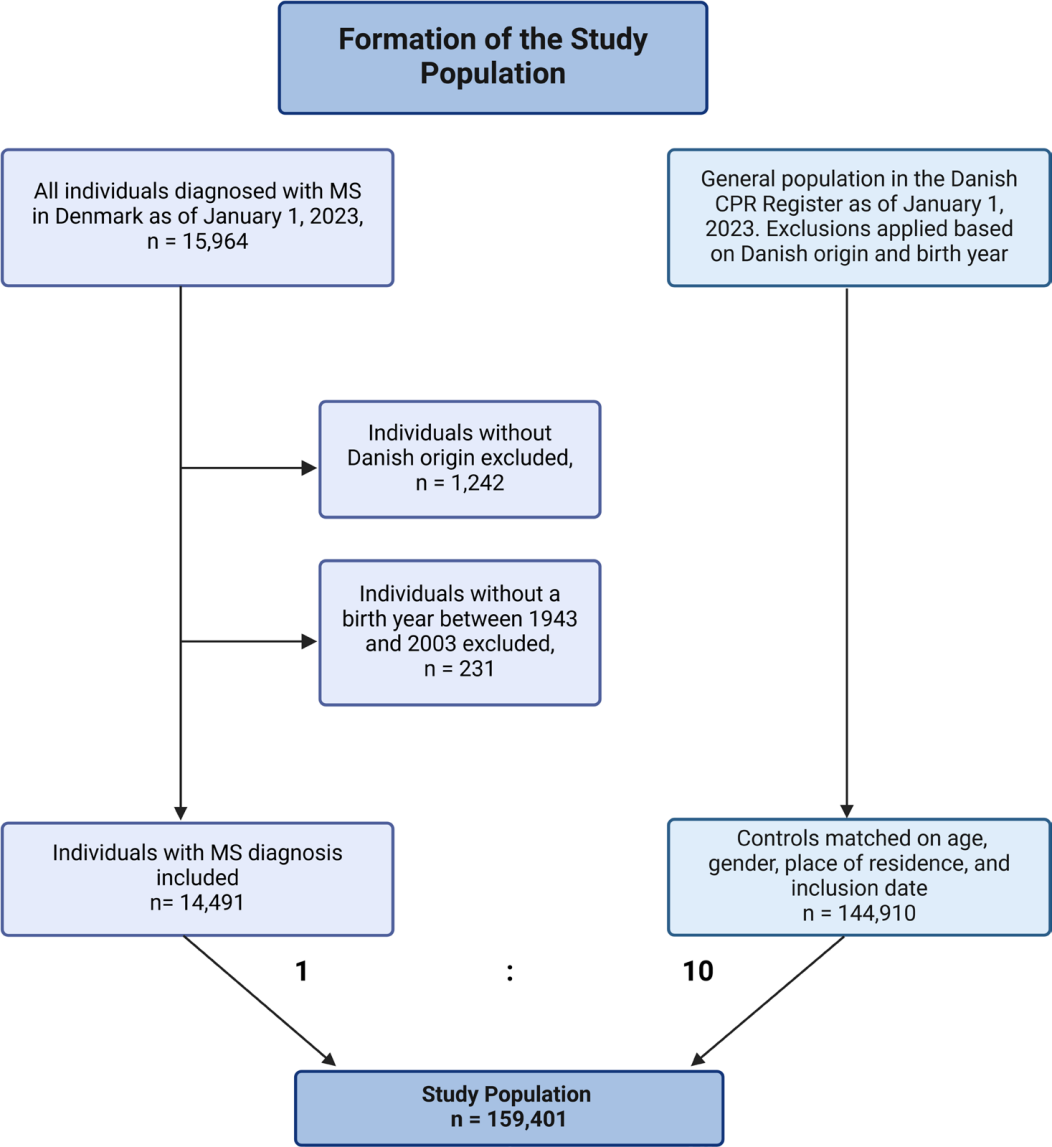
Flowchart illustrating the formation of the study population. CPR, Central Person Register; MS, multiple sclerosis. Figure created in https://BioRender.com.

Every resident in Denmark has a unique civil registration number used for all healthcare system interactions [11, 12]. The Danish Multiple Sclerosis Registry, dating back to 1956, includes data on all patients with MS in Denmark, such as sex, age, clinical characteristics, and treatment information [13]. In the present study, we included all individuals with MS who were between 20 and 80 years old and alive on January 1, 2023, and followed them for 1 year. We obtained baseline data on age, sex, years since MS diagnosis, Expanded Disability Status Scale score (taken within a maximum of 2 years from the baseline) [14], residence type, country of origin, and previous use of disease‐modifying therapies (DMTs) for study participants. For each included individual with MS, 10 matched controls were randomly selected from the general Danish population based on age, sex, place of residence, and baseline date using the Civil Registration System [15]. All included control individuals were confirmed to be alive as of January 1, 2023. For further information regarding the formation of the study population (Figure 1).

The Danish National Prescription Registry is a comprehensive database that systematically records all prescription medications dispensed at outpatient pharmacies by all people residing in Denmark classified under the Anatomical Therapeutic Chemical (ATC) system [11]. Notably, over‐the‐counter medications and hospital‐administered drugs are not included in this registry. For this study, we extracted data on all prescriptions collected for each of our study participants during the year 2023. We collected data on medication use at ATC level 2 (drug group) and 5 (specific drug). An individual was defined as a user of a medication if they redeemed one or more prescriptions for the substance during 2023. Similarly, an individual was categorized as a user of a drug group if they redeemed one or more prescriptions within that group. We have data for the ATC groups A (alimentary tract and metabolism), B (blood and blood‐forming organs), C (cardiovascular system), D (dermatologicals), G (genitourinary system and sex hormones), H (systemic hormonal preparations), J (anti‐infectives for systemic use), M (musculoskeletal system), N (nervous system), R (respiratory system), and S (sensory organs), including all subcodes. Medications that were used significantly more or less frequently by MS patients compared to the general population were analyzed separately. Additionally, the most used drugs among MS patients as well as the most pronounced differences between MS patients and controls were identified and examined in more detail.

### 3.2 Anonymity Regulations

Due to data protection regulations, we report only on medications used by at least five individuals in both the MS and control groups.

### 3.3 Statistical Approach

Statistics were applied for the Poisson distribution. Relative risks (RRs) along with 95% CIs were calculated to evaluate the association between medication usage among individuals with MS and the general population. The Bonferroni correction was applied to all associations to adjust for multiple testing, thereby minimizing the risk of false positives and ensuring that only the most statistically significant signals were highlighted. To identify overall trends as well as specific medications, we analyzed medication use at both ATC level 2 (therapeutic classes) and level 5 (specific drugs).

Sensitivity analyses were conducted at ATC level 2 to assess the robustness of the results. These included stratified analyses to determine whether differences in dispensed prescription drugs between MS patients and controls differed by sex and age. Finally, a sensitivity analysis was conducted to examine the use of hormonal contraceptives in females below 50 years.

A two‐sided *p*‐value of < 0.05 was considered statistically significant, and all statistical analyses were conducted using SAS software (SAS Institute Inc., Cary, NC).

## 4 Results

### 4.1 Study Population and Medication Use

This study analyzed the medication usage of 14,491 MS patients in 2023 and compared it to data from 144,910 matched controls (Figure 1). The baseline characteristics of the study population are detailed in Table 1. During this one‐year follow‐up, 172 individuals with MS and 815 controls died.

**TABLE 1:** Characteristics of the study population at baseline.

Characteristic	Individuals with MS (*n* = 14,491)	Control group (*n* = 144,910)
Age, years		
Mean (SD)	52.49 (13.02)	52.49 (13.02)
Sex		
Female, *n* (%)	9945 (68.63)	99,450 (68.63)
Male, *n* (%)	4546 (31.37)	45,460 (31.37)
Years since MS diagnosis		
Mean (SD)	13.44 (9.42)	—
EDSS score categories		
< 4, *n* (%)	7858 (54.23)	—
4–5.5, *n* (%)	1503 (10.37)	—
≥ 6, *n* (%)	2062 (14.23)	—
Residence type		
Capital municipality, *n* (%)	3467 (23.93)	34,670 (23.93)
Rural municipality, *n* (%)	3143 (21.69)	31,430 (21.69)
Suburban municipality, *n* (%)	2619 (18.07)	26,190 (18.07)
Provincial municipality, *n* (%)	3606 (24.88)	36,060 (24.88)
Metropolitan municipality, *n* (%)	1656 (11.43)	16,560 (11.43)
Country of origin		
Denmark, *n* (%)	14,491 (100)	144,910 (100)

*Note:* The baseline date was January 1, 2023, for both cases and controls. The most recent EDSS score, taken within a maximum of 2 years from the baseline, was included for each patient.

Abbreviations: EDSS, Expanded Disability Status Scale; MS, multiple sclerosis; SD, standard deviation.

An overview of DMTs for MS patients at baseline and prior to inclusion is presented in Table 2. A total of 3148 patients (21.72%) never received any DMT, while the rest had varied exposure to different disease‐modifying drugs, with the most common being interferon beta‐1a, teriflunomide, and dimethyl fumarate.

**TABLE 2:** Disease‐modifying therapy.

Disease‐modifying therapy	Number of users with MS (%)
Alemtuzumab	390 (2.69)
Cladribine	624 (4.31)
Dimethyl fumarate	3485 (24.05)
Fampridine	1088 (7.51)
Fingolimod	2335 (16.11)
Glatiramer acetate	2610 (18.01)
Human IgG	171 (1.18)
Interferon beta‐1a	6900 (47.62)
Interferon beta‐1b	1075 (7.42)
Methylprednisolone	161 (1.11)
Mitoxantrone	375 (2.59)
Natalizumab	3054 (21.08)
Ocrelizumab	1845 (12.73)
Ofatumumab	251 (1.73)
Peginterferon beta‐1a	530 (3.66)
Rituximab	515 (3.55)
Teriflunomide	3603 (24.86)

*Note:* A summary of the types of disease‐modifying therapies (DMT) used by multiple sclerosis (MS) patients, *n* = 14.491, at baseline in the study and any prior DMT treatments. A total of 3148 patients (21.72%) had not received any DMT.

The analysis of medication use, excluding DMTs, focused on identifying drug groups with significantly increased or decreased use among MS patients compared to the general population.

**FIGURE 2:**
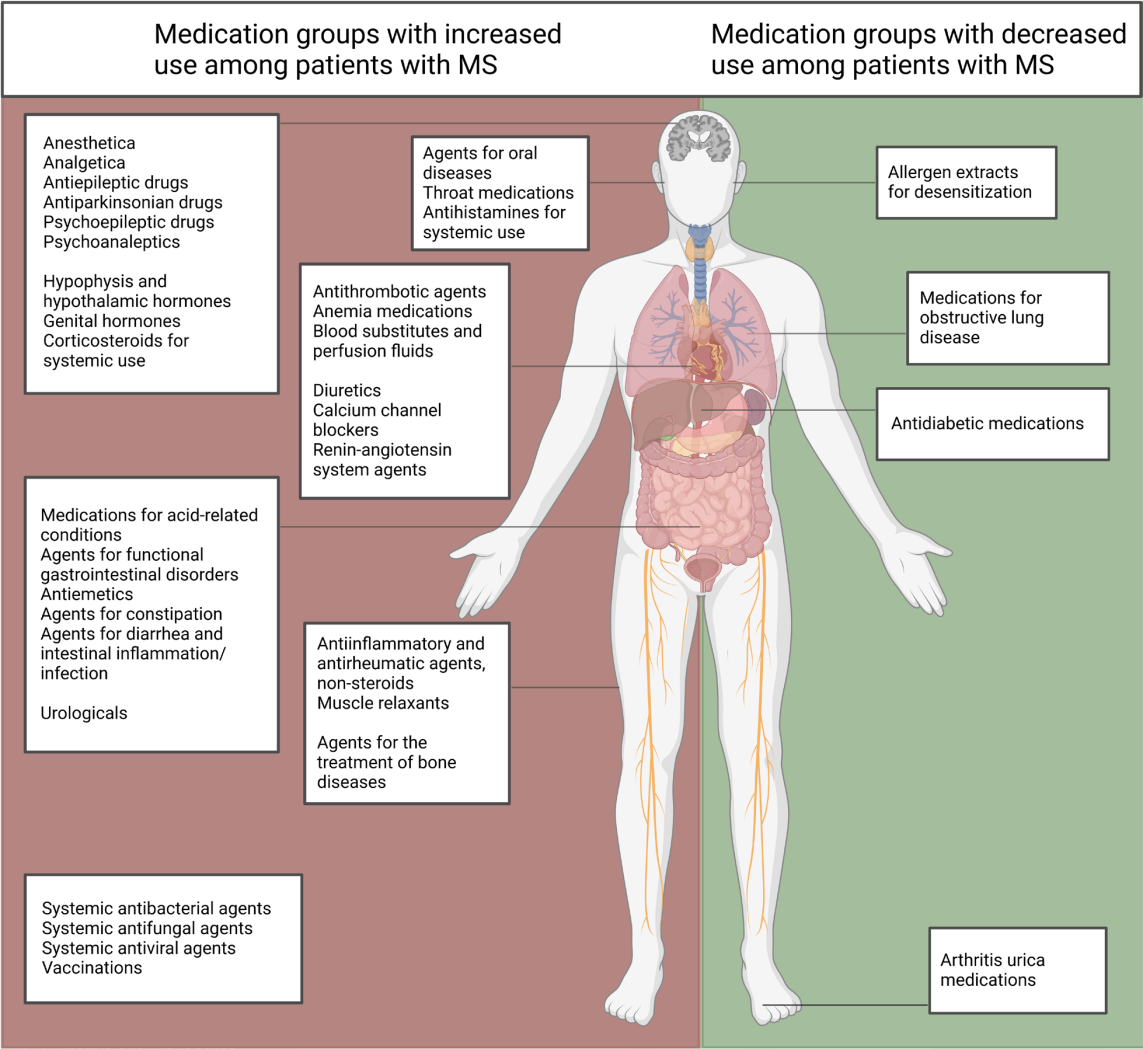
Medication groups with increased (left side) and decreased (right side) use among patients with MS. Only associations significant after adjustment for multiple testing with Bonferroni correction are illustrated. For specific counts, relative risks and confidence intervals within drug groups of medication please see Table 3. Topical agents are not included in the overview. Figure created in https://BioRender.com. MS, multiple sclerosis.

This study shows that patients living with MS have a significantly higher medication consumption compared to the general population. Medication consumption is elevated across nearly all disease and ATC domains, including neuropharmacological, musculoskeletal, urological, and cardiovascular drugs, as well as spasmolytics and various antibiotics. In a few certain therapeutic drug groups, we observed lower consumption of drugs compared to the background population, including antidiabetic drugs and therapies aimed at gout, obstructive lung disease, diabetes, and allergen desensitization (Figure 2) and (Table 3).

**TABLE 3:** Medication use among MS cases and controls.

Therapeutic classes	Users among MS cases, *n* (%)	Users among controls, *n* (%)	Relative risk (95% CI)
Increased use among patients with MS			
Anesthetica	66 (0.46)	236 (0.16)	2.80 (2.13–3.67)
Analgetica	7457 (51.46)	47,817 (33.00)	1.56 (1.52–1.60)
Antiepileptic drugs	949 (6.55)	3254 (2.25)	2.92 (2.71–3.14)
Antiparkinsonian drugs	353 (2.44)	1480 (1.02)	2.39 (2.12–2.68)
Psychoepileptic drugs	2081 (14.36)	12,519 (8.64)	1.66 (1.59–1.74)
Psychoanaleptics	3809 (26.29)	17,965 (12.40)	2.12 (2.05–2.20)
Hypophysis and hypothalamic hormones	104 (0.72)	388 (0.27)	2.68 (2.16–3.33)
Genital hormones	2279 (15.73)	18,120 (12.50)	1.26 (1.20–1.31)
Corticosteroids for systemic use	615 (4.24)	4694 (3.24)	1.31 (1.20–1.43)
Agents for oral diseases	695 (22.34)	3237 (2.23)	2.15 (1.98–2.33)
Throat medications	27 (0.19)	123 (0.08)	2.20 (1.45–3.33)
Antihistamines for systemic use	1272 (8.78)	11,143 (7.69)	1.14 (1.08–1.21)
Antithrombotic agents	1597 (11.02)	13,641 (9.41)	1.17 (1.11–1.23)
Anemia medications	993 (6.85)	6701 (4.62)	1.48 (1.39–1.58)
Blood substitutes and perfusion fluids	7 (0.05)	11 (0.01)	6.36 (2.47–16.42)
Diuretics	1657 (11.43)	12,357 (8.53)	1.34 (1.27–1.41)
Calcium channel blockers	1734 (11.97)	15,882 (10.96)	1.09 (1.04–1.15)
Renin‐angiotensin system agents	2958 (20.41)	26,883 (18.55)	1.10 (1.06–1.14)
Medications for acid‐related conditions	2764 (19.07)	21,535 (14.86)	1.28 (1.23–1.34)
Agents for functional gastrointestinal disorders	323 (2.23)	1798 (1.24)	1.80 (1.60–2.02)
Antiemetics	179 (1.24)	854 (0.59)	2.10 (1.78–2.46)
Agents for constipation	1779 (12.28)	3857 (2.66)	4.61 (4.36–4.88)
Agents for diarrhea and intestinal inflammation/infection	403 (2.78)	2724 (1.88)	1.48 (1.33–1.64)
Urologicals	3019 (20.83)	6045 (4.17)	4.99 (4.78–5.22)
Anti‐inflammatory and antirheumatic agents, non‐steroids	2994 (20.66)	25,167 (17.37)	1.19 (1.15–1.24)
Muscle relaxants	2864 (19.76)	3631 (2.51)	7.89 (7.51–8.28)
Agents for the treatment of bone diseases	814 (5.62)	3435 (2.37)	2.37 (2.20–2.56)
Systemic antibacterial agents	5307 (36.62)	36,198 (24.98)	1.47 (1.42–1.51)
Systemic antifungal agents	426 (2.94)	3212 (2.22)	1.33 (1.20–1.47)
Systemic antiviral agents	803 (5.54)	5165 (3.56)	1.55 (1.44–1.67)
Vaccinations	355 (2.45)	1800 (1.24)	1.97 (1.76–2.21)
Decreased use among patients with MS			
Allergen extracts for desensitization	12 (0.08)	418 (0.29)	0.29 (0.16–0.51)
Medications for obstructive lung disease	1320 (9.11)	14,721 (10.16)	0.90 (0.85–0.95)
Antidiabetic medications	1257 (8.67)	14,774 (10.20)	0.85 (0.80–0.90)
Arthritis urica medications	101 (0.70)	1928 (1.33)	0.52 (0.43–0.64)

*Note:* Medication groups used more or less frequently by MS patients compared to the general population. Only associations significant after adjustment for multiple testing with Bonferroni correction are listed. Topical agents are not included in the overview.

Abbreviation: MS, multiple sclerosis.

Notable findings include a higher use of analgesic and antiepileptic drugs, as well as antiparkinsonian drugs, among individuals with MS compared to controls. Analgetica were used by 51% of individuals with MS versus 33% of controls, yielding an RR of 1.56 (95% CI: 1.52–1.60). Furthermore, agents for constipation showed significantly higher usage in individuals with MS (12%) compared to controls (3%), with an RR of 4.61 (95% CI: 4.36–4.88). When analyzing specific drugs at ATC level 5, we identified the medications with the most significant differences between individuals with MS and controls, with the highest RR observed for modafinil (RR: 323.33, 95% CI: 228.67–457.19), baclofen (RR: 36.51, 95% CI: 33.33–39.99), and fesoterodine (RR: 36.36, 95% CI: 18.66–70.87) compared to controls. For a complete list of associations within specific drugs, please see Table S1.

### 4.2 Differences Between MS Patients and Controls by Sex

In the sensitivity analysis focusing on potential biological sex differences, the same general trends were observed among females and males as in the overall group. However, some signals were no longer strong enough to remain significant after Bonferroni correction. For example, only antidiabetics and allergen extracts for desensitization continued to be less commonly used among females, whereas males were exclusively treated less frequently with medication against arthritis urica. For further details, see supplement material Tables S2 and S3.

### 4.3 Differences Between MS Patients and Controls by Age

Stratified analyses by age (< 50 vs. ≥ 50 years) revealed that while overall patterns were largely consistent across age groups, differences were more notable among younger individuals for specific medication categories, including anti‐inflammatory treatments, vaccinations, antifungal agents, and selected antihypertensives. Notably, in all of these categories, individuals with MS exhibited significantly higher use compared to matched controls in the overall cohort and in the younger age group, whereas associations were not statistically significant among older individuals.

### 4.4 Systemic Hormonal Contraceptives

In females below 50 years, 682 women with MS and 6113 women without MS redeemed at least one prescription for hormonal contraceptives in 2023, corresponding to a relative risk of 1.12 (95% CI: 1.03–1.21) among MS patients.

## 5 Discussion

This nationwide study examined differences in medication use between MS patients and the general population. Our findings revealed extensive use of various therapeutic classes and specific drugs, including treatments for well‐known MS‐related comorbidities as well as newly identified associations.

Considering the pathophysiology of MS, it is expected that these patients exhibit an increased use of both spasmolytic and urological medications compared to controls, as observed in our study and supported by the current literature [16, 17, 18]. A Swedish nationwide study reported that 10%–19% of MS patients received baclofen, typically within the first 6 months to 3 years following diagnosis. The strongest predictors of baclofen initiation were higher disability (EDSS ≥ 6) and younger age [16]. Furthermore, we observed a high use of painkillers, including opioids, NSAIDs, and paracetamol. This is probably attributed to both neuropathic pain as well as pain resulting from immobilization as the disease progresses, aligning with findings from previous studies [19, 20, 21]. A large Swedish study found a strong association between MS and the use of pain treatment, with an even stronger association for neuropathic pain specifically [22]. These findings support our observation of substantially elevated use of antiepileptic drugs, which are commonly prescribed for this pain type.

However, NSAIDs and paracetamol are also used to manage side effects associated with certain DMTs [23]. Furthermore, the immunosuppressive properties of DMTs, along with immobilization and bladder dysfunction, naturally increase the risk of infections, leading to a higher use of antibacterial, antiviral, and antifungal agents [24, 25]. We also found a slightly higher use of hormonal contraception among women with MS compared to controls, highlighting the need for family planning in this group.

In summary, our results are in accordance with other large‐scale MS cohorts, both in terms of the direction and magnitude of associations for key symptomatic drug classes. This strengthens the external validity of our findings and underscores the consistent, substantial medication burden associated with MS.

Interestingly, our study also identified several drug classes that were less commonly used in the MS population compared to controls without MS. These included, among others, antidiabetic medications and treatments for chronic obstructive pulmonary disease (COPD). While speculative, this pattern may reflect lifestyle changes following an MS diagnosis—such as the strongly recommended smoking cessation—potentially leading to a reduced risk of COPD. Notably, a Danish nationwide study found a significantly reduced incidence of asthma and COPD after MS onset, supporting the concept of inverse comorbidity [26]. However, these findings may also reflect underdiagnosis of COPD in MS patients, potentially due to reduced physical activity, or be influenced by healthy survivor bias. These findings highlight the need for more systematic symptom screening in MS care, particularly for common but often under‐recognized symptoms such as pain, spasticity, and constipation. Moreover, the unexpectedly lower use of antidiabetic medications among MS patients raises important mechanistic questions that warrant further investigation, including possible differences in diagnostic thresholds, metabolic pathways, or healthcare‐seeking behavior in this population.

Furthermore, this study confirmed a low prevalence of anti‐gout medication among MS patients, with recent findings indicating that its use was already reduced several years before MS diagnosis [27].

## 6 Strengths and Limitations

The current study presents a nationwide analysis of medication use among individuals with MS, utilizing extensive coding and a complete population dataset. A key strength of the study lies in the inclusion of an adequate control group, which enhances the reliability and comparability of the findings. Furthermore, the analysis offers a valuable contemporary representation of medication usage, ranging from therapeutic classes to specific drugs, providing detailed insights into treatment patterns.

Despite these strengths, certain limitations must be considered. Generalizability may be restricted due to the unique characteristics of the Danish and similar Nordic healthcare systems. Additionally, our analysis focuses exclusively on prescription medications, which may introduce limitations, as hospital‐administered drugs, over‐the‐counter treatments, and non‐prescription therapies were not considered. Moreover, we report only the most robust associations that remained significant after the Bonferroni correction. While this stringent approach minimizes the risk of false‐positive findings, it may also overlook weaker but potentially meaningful associations. Future research could explore these subtler signals to provide a more comprehensive understanding of medication use in MS, and given the cross‐sectional nature of the present study, it will be crucial for subsequent investigations to explore the temporality of MS disease course and medication use and examine actual causal associations within these populations through designs specifically tailored for causal inference.

Conclusively, this study provides a detailed analysis of medication usage among individuals with MS in Denmark in a large, nationwide setting. The findings underscore the wide range of pharmacological treatments used to manage the diverse symptoms and comorbidities associated with MS. This information could be used by healthcare providers to enhance patient care strategies and target areas in need of attention within the MS patient population.

## Author Contributions

J.W.M., H.H., M.M., E.F., and I.M.H. contributed to the conception and design of the study. H.H. performed the data analysis. J.W.M. drafted the manuscript. M.M., E.F., and I.M.H. supervised the project and contributed to the interpretation of results. All authors revised the manuscript critically for important intellectual content and approved the final version.

## Conflicts of Interest

M.M. has served on the scientific advisory board for Sanofi, Novartis, Merck, and Moderna and has received honoraria for lecturing from Biogen, Merck, Novartis, Roche, Sanofi, Moderna, and Neuroxpharm. The rest of the authors declare no conflicts of interest.

## Supporting information


**Table S1:** Overview of differences in medication use at specific drug level (ATC level 5) between individuals with MS (*n* = 14,491) and controls (*n* = 144,910). Only statistically significant associations after adjustment for multiple testing with Bonferroni correction are listed.
**Table S2:** Medication groups with increased and decreased use among females with MS (*n* = 9945) compared to females in the control group (*n* = 99,450). Only associations significant after adjustment for multiple testing with Bonferroni correction are listed. Topical agents are not included in the overview. ATC, anatomical therapeutic chemical classification system; CI, confidence interval; MS, multiple sclerosis.
**Table S3:** Medication groups with increased and decreased use among males with MS (*n* = 4546) compared to males in the control group (*n* = 45,460). Only associations significant after adjustment for multiple testing with Bonferroni correction are listed. Topical agents are not included in the overview. ATC, anatomical therapeutic chemical classification system; CI, confidence interval; MS, multiple sclerosis.

## Data Availability

The data underlying this article cannot be shared publicly due to data protection regulation. All data are stored in a protected server environment at Statistics Denmark and can be accessed only by researchers authorized by Statistics Denmark and approved by the Danish Multiple Sclerosis Registry board.
