# Supplementary material for: Medication Use in Multiple Sclerosis: A Population‐Based Comparison With the General Danish Population
Source: Ann Clin Transl Neurol. 2025 Jul 31;12(11):2206–13. doi: 10.1002/acn3.70155 (PMC12623830; doi:10.1002/acn3.70155)
Supplement: Supplementary file 1 — Table S1: Overview of differences in medication use at specific drug level (ATC level 5) between individuals with MS (n = 14,491) and controls (n = 144,910). Only statistically significant associations after adjustment for multiple testing with Bonferroni correction are listed. Table S2: Medication groups with increased and decreased use among females with MS (n = 9945) compared to females in the control group (n = 99,450). Only associations significant after adjustment for multiple testing with Bonferroni correction are listed. Topical agents are not included in the overview. ATC, anatomical therapeutic chemical classification system; CI, confidence interval; MS, multiple sclerosis. Table S3: Medication groups with increased and decreased use among males with MS (n = 4546) compared to males in the control group (n = 45,460). Only associations significant after adjustment for multiple testing with Bonferroni correction are listed. Topical agents are not included in the overview. ATC, anatomical therapeutic chemical classification system; CI, confidence interval; MS, multiple sclerosis. [file ACN3-12-2206-s001.docx]

**Online Supplement - Patterns of Medication Use in the Danish Population with Multiple Sclerosis**

Josefine Windfeld-Mathiasen^1,2,3^, Henrik Horwitz^1,2^, Ida M. Heerfordt^1,4^, Elisabeth Framke^3^, Melinda Magyari^2,3,5^

^1^Department of Clinical Pharmacology, Copenhagen University Hospital - Bispebjerg and Frederiksberg, Denmark

^2^Department of Clinical Medicine, University of Copenhagen, Denmark

^3^Danish Multiple Sclerosis Registry, Department of Neurology, Copenhagen University Hospital - Rigshospitalet, Glostrup, Denmark

^4^Department of Geriatric and Palliative Medicine, Copenhagen University Hospital - Bispebjerg and Frederiksberg, Denmark

^5^Danish Multiple Sclerosis Center, Department of Neurology, Copenhagen University Hospital - Rigshospitalet, Glostrup, Denmark

**eResults**

Differences in medication use at specific drug level (ATC level 5) are presented in Table S1.

Sensitivity analyses of drug groups (ATC level 2) stratified by sex, are detailed in Tables S2-S3.

| **ATC-group** | **Drug** | **Users among MS cases** | **Users among controls** | **Relative risk (95% CI)** |
| --- | --- | --- | --- | --- |
|  |  |  |  |  |
|  |  | N (%) | N (%) |  |
|  |  |  |  |  |
|  |  |  |  |  |
| **A: Alimentary tract and metabolism** | | |  |  |
|  | Semaglutide | 681 (4.70) | 8,769 (6.05) | 0.78 (0.72-0.84) |
|  | Pantoprazole | 1,566 (10.81) | 13,357 (9.22) | 1.17 (1.11-1.24) |
|  | Lansoprazole | 493 (3.40) | 3,743 (2.58) | 1.32 (1.20-1.45) |
|  | Nystatin | 133 (0.92) | 916 (0.63) | 1.45 (1.21-1.74) |
|  | Ondansetron | 124 (0.86) | 795 (0.55) | 1.56 (1.29-1.88) |
|  | Metoclopramide | 245 (1.69) | 1,425 (0.98) | 1.72 (1.50-1.97) |
|  | Potassium chloride | 1,045 (7.21) | 5,684 (3.92) | 1.84 (1.72-1.96) |
|  | Sodium fluoride | 595 (4.11) | 2,504 (1.73) | 2.38 (2.17-2.60) |
|  | Bisacodyl | 365 (2.52) | 1,422 (0.98) | 2.57 (2.29-2.88) |
|  | Magnesium hydroxide | 405 (2.79) | 1,544 (1.07) | 2.62 (2.35-2.93) |
|  | Loperamide | 134 (0.92) | 473 (0.33) | 2.83 (2.34-3.43) |
|  | Glycopyrronium bromide | 28 (0.19) | 89 (0.06) | 3.15 (2.06-4.81) |
|  | Lactulose | 100 (0.69) | 309 (0.21) | 3.24 (2.58-4.05) |
|  | Sodium chloride | 26 (0.18) | 58 (0.04) | 4.48 (2.82-7.12) |
|  | Sodium picosulfate | 347 (2.39) | 708 (0.49) | 4.9 (4.31-5.57) |
|  | Macrogol, combinations | 901 (6.22) | 1,754 (1.21) | 5.14 (4.74-5.57) |
|  | Prucalopride | 43 (0.30) | 82 (0.06) | 5.24 (3.63-7.58) |
|  | Vitamin B-complex, incl. combinations | 49 (0.34) | 90 (0.06) | 5.44 (3.84-7.71) |
|  | Scopolamine | 20 (0.14) | 33 (0.02) | 6.06 (3.48-10.56) |
|  | Atropine | 9 (0.06) | 13 (0.01) | 6.92 (2.96-16.20) |
|  | Glycerol | 25 (0.17) | 18 (0.01) | 13.89 (7.58-25.46) |
|  | Dronabinol | 40 (0.28) | 28 (0.02) | 14.29 (8.81-23.15) |
|  | Sodium lauryl sulfoacetate, incl. combinations | 455 (3.14) | 225 (0.16) | 20.22 (17.24-23.72) |
|  | Docusate sodium, incl. combinations | 534 (3.69) | 208 (0.14) | 25.67 (21.87-30.13) |
|  |  |  |  |  |
| **B: Blood and blood forming organs** | |  |  |  |
|  | Acetylsalicylic acid | 774 (5.34) | 6,213 (4.29) | 1.25 (1.16-1.34) |
|  | Cyanocobalamin | 566 (3.91) | 3,769 (2.60) | 1.5 (1.37-1.64) |
|  | Ferrous sulfate | 137 (0.95) | 872 (0.60) | 1.57 (1.31-1.88) |
|  | Hydroxocobalamin | 174 (1.20) | 1,050 (0.72) | 1.66 (1.41-1.95) |
|  |  |  |  |  |
| **C: Cardiovascular system** | | |  |  |
|  | Bendroflumethiazide and potassium | 844 (5.82) | 7,118 (4.91) | 1.19 (1.10-1.27) |
|  | Furosemide | 647 (4.46) | 3,676 (2.54) | 1.76 (1.62-1.91) |
|  | Colestyramine | 62 (0.43) | 236 (0.16) | 2.63 (1.99-3.48) |
|  |  |  |  |  |
| **D: Dermatologicals** | |  |  |  |
|  | Mometasone | 423 (2.92) | 3,438 (2.37) | 1.23 (1.11-1.36) |
|  | Hydrocortisone butyrate | 594 (4.10) | 4,805 (3.32) | 1.24 (1.14-1.35) |
|  | Fusidic acid | 312 (2.15) | 2,275 (1.57) | 1.37 (1.22-1.54) |
|  | Imidazoles/triazoles in combination with corticosteroids | 598 (4.13) | 4,015 (2.77) | 1.49 (1.37-1.62) |
|  | Ketoconazole | 395 (2.73) | 2,396 (1.65) | 1.65 (1.48-1.83) |
|  | Miconazole | 228 (1.57) | 919 (0.63) | 2.48 (2.15-2.87) |
|  | Tars | 24 (0.17) | 50 (0.03) | 4.80 (2.95-7.81) |
|  |  |  |  |  |
| **G: Genito urinary system and sex hormones** | | |  |  |
|  | Estradiol | 1,400 (9.66) | 10,121 (6.98) | 1.38 (1.31-1.46) |
|  | Sildenafil | 609 (4.20) | 2,876 (1.98) | 2.12 (1.94-2.31) |
|  | Tamsulosin | 212 (1.46) | 993 (0.69) | 2.13 (1.84-2.48) |
|  | Alfuzosin | 96 (0.66) | 447 (0.31) | 2.15 (1.72-2.68) |
|  | Tadalafil | 273 (1.88) | 911 (0.63) | 3 (2.62-3.43) |
|  | Alprostadil | 52 (0.36) | 64 (0.04) | 8.13 (5.64-11.71) |
|  | Solifenacin | 284 (1.96) | 266 (0.18) | 10.68 (9.03-12.62) |
|  | Trospium | 30 (0.21) | 17 (0.01) | 17.65 (9.73-31.99) |
|  | Mirabegron | 1,110 (7.66) | 620 (0.43) | 17.9 (16.23-19.75) |
|  | Tolterodine | 858 (5.92) | 403 (0.28) | 21.29 (18.91-23.97) |
|  | Fesoterodine | 40 (0.28) | 11 (0.01) | 36.36 (18.66-70.87) |
|  |  |  |  |  |
| **H: Systemic hormonal preparations, excl. sex hormones and insulins** | |  |  |  |
|  | Thiamazole | 126 (0.87) | 691 (0.48) | 1.82 (1.51-2.20) |
|  | Methylprednisolone | 216 (1.49) | 394 (0.27) | 5.48 (4.64-6.47) |
|  | Desmopressin | 84 (0.58) | 101 (0.07) | 8.32 (6.23-11.11) |
|  |  |  |  |  |
| **J: Anti-infectives for systemic use** | |  |  |  |
|  | Phenoxymethylpenicillin | 1,946 (13.43) | 16,915 (11.67) | 1.15 (1.10-1.21) |
|  | Aciclovir | 465 (3.21) | 3,520 (2.43) | 1.32 (1.20-1.46) |
|  | Fluconazole | 411 (2.84) | 3,098 (2.14) | 1.33 (1.20-1.47) |
|  | Roxithromycin | 298 (2.06) | 2,135 (1.47) | 1.4 (1.24-1.58) |
|  | Dicloxacillin | 771 (5.32) | 5,281 (3.64) | 1.46 (1.35-1.57) |
|  | Valaciclovir | 276 (1.90) | 1,694 (1.17) | 1.63 (1.43-1.85) |
|  | Amoxicillin and beta-lactamase inhibitor | 551 (3.80) | 3,247 (2.24) | 1.70 (1.55-1.86) |
|  | Amoxicillin | 552 (3.81) | 2,872 (1.98) | 1.92 (1.75-2.11) |
|  | Zoster, purified antigen | 61 (0.42) | 255 (0.18) | 2.39 (1.81-3.16) |
|  | Pivmecillinam | 2,228 (15.38) | 8,609 (5.94) | 2.59 (2.47-2.71) |
|  | Ciprofloxacin | 363 (2.51) | 1,225 (0.85) | 2.96 (2.64-3.33) |
|  | Sulfamethizole | 426 (2.94) | 1,182 (0.82) | 3.60 (3.23-4.03) |
|  | Ampicillin | 34 (0.23) | 88 (0.06) | 3.86 (2.60-5.74) |
|  | Pivampicillin | 48 (0.33) | 117 (0.08) | 4.10 (2.93-5.74) |
|  | Pneumococcus, purified polysaccharides antigen conjugated | 56 (0.39) | 133 (0.09) | 4.21 (3.08-5.75) |
|  | Cefuroxime | 20 (0.14) | 38 (0.03) | 5.26 (3.06-9.04) |
|  | Pneumococcus, purified polysaccharides antigen | 155 (1.07) | 254 (0.18) | 6.1 (5.00-7.45) |
|  | Trimethoprim | 586 (4.04) | 948 (0.65) | 6.18 (5.58-6.85) |
|  | Nitrofurantoin | 218 (1.50) | 318 (0.22) | 6.86 (5.77-8.14) |
|  | Methenamine | 128 (0.88) | 87 (0.06) | 14.71 (11.21-19.32) |
|  | Nirmatrelvir and ritonavir | 85 (0.59) | 49 (0.03) | 17.35 (12.21-24.65) |
|  |  |  |  |  |
| **M: Musculo-sceletal system** | | |  |  |
|  | Allopurinol | 76 (0.52) | 1627 (1.12) | 0.47 (0.37-0.59) |
|  | Ibuprofen | 2,697 (18.61) | 22,240 (15.35) | 1.21 (1.17-1.26) |
|  | Chlorzoxazone | 367 (2.53) | 2,833 (1.96) | 1.30 (1.16-1.44) |
|  | Diclofenac | 64 (0.44) | 302 (0.21) | 2.12 (1.62-2.78) |
|  | Alendronic acid | 686 (4.73) | 3,014 (2.08) | 2.28 (2.09-2.47) |
|  | Denosumab | 120 (0.83) | 389 (0.27) | 3.08 (2.51-3.79) |
|  | Tizanidine | 974 (6.72) | 355 (0.24) | 27.44 (24.30-30.98) |
|  | Baclofen | 2,154 (14.86) | 590 (0.41) | 36.51 (33.33-39.99) |
|  |  |  |  |  |
| **N: Nervous system** | |  |  |  |
|  | Sertraline | 646 (4.46) | 5,285 (3.65) | 1.22 (1.13-1.33) |
|  | Oxycodone | 293 (2.02) | 2,267 (1.56) | 1.29 (1.14-1.46) |
|  | Oxazepam | 256 (1.77) | 1,763 (1.22) | 1.45 (1.27-1.66) |
|  | Paracetamol | 6,161 (42.52) | 41,867 (28.89) | 1.47 (1.43-1.51) |
|  | Morphine | 700 (4.83) | 4,701 (3.24) | 1.49 (1.38-1.61) |
|  | Melatonin | 579 (4.00) | 3,875 (2.67) | 1.49 (1.37-1.63) |
|  | Mirtazapine | 378 (2.61) | 2,346 (1.62) | 1.61 (1.45-1.80) |
|  | Naltrexone | 88 (0.61) | 544 (0.38) | 1.62 (1.29-2.03) |
|  | Escitalopram | 143 (0.99) | 876 (0.60) | 1.63 (1.37-1.95) |
|  | Tramadol | 589 (4.06) | 3,433 (2.37) | 1.72 (1.57-1.87) |
|  | Eletriptan | 78 (0.54) | 454 (0.31) | 1.72 (1.35-2.18) |
|  | Lamotrigine | 341 (2.35) | 1,865 (1.29) | 1.83 (1.63-2.05) |
|  | Nicotine | 51 (0.35) | 274 (0.19) | 1.86 (1.38-2.51) |
|  | Nortriptyline | 97 (0.67) | 481 (0.33) | 2.02 (1.62-2.51) |
|  | Duloxetine | 513 (3.54) | 2,361 (1.63) | 2.17 (1.97-2.39) |
|  | Zolpidem | 315 (2.17) | 1,445 (1.00) | 2.18 (1.93-2.46) |
|  | Codeine and acetylsalicylic acid | 100 (0.69) | 442 (0.31) | 2.26 (1.82-2.81) |
|  | Midazolam | 70 (0.48) | 295 (0.20) | 2.37 (1.83-3.08) |
|  | Zopiclone | 608 (4.20) | 2,422 (1.67) | 2.51 (2.30-2.74) |
|  | Alprazolam | 111 (0.77) | 420 (0.29) | 2.64 (2.14-3.26) |
|  | Diazepam | 138 (0.95) | 522 (0.36) | 2.64 (2.19-3.19) |
|  | Buprenorphine | 58 (0.40) | 218 (0.15) | 2.66 (1.99-3.55) |
|  | Citalopram | 642 (4.43) | 2,398 (1.65) | 2.68 (2.45-2.92) |
|  | Levetiracetam | 121 (0.84) | 449 (0.31) | 2.69 (2.20-3.29) |
|  | Lidocaine | 54 (0.37) | 197 (0.14) | 2.74 (2.03-3.70) |
|  | Pramipexole | 294 (2.03) | 1,060 (0.73) | 2.77 (2.44-3.16) |
|  | Pregabalin | 737 (5.09) | 2,514 (1.73) | 2.93 (2.70-3.18) |
|  | Fentanyl | 70 (0.48) | 238 (0.16) | 2.94 (2.25-3.84) |
|  | Acetylsalicylic acid, combinations excl. psycholeptics | 56 (1.08) | 491 (0.34) | 3.18 (2.65-3.80) |
|  | Clonazepam | 80 (0.55) | 248 (0.17) | 3.23 (2.51-4.15) |
|  | Gabapentin | 1,699 (11.72) | 4,347 (3.00) | 3.91 (3.70-4.13) |
|  | Amitriptyline | 567 (3.91) | 1,376 (0.95) | 4.12 (3.74-4.54) |
|  | Amantadine | 17 (0.12) | 22 (0.02) | 7.73 (4.10-14.55) |
|  | Carbamazepine | 195 (1.35) | 212 (0.15) | 9.2 (7.57-11.17) |
|  | Cannabidiol | 9 (0.06) | 8 (0.01) | 11.25 (4.34-29.16) |
|  | Oxcarbazepine | 218 (1.5) | 124 (0.09) | 17.58 (14.10-21.92) |
|  | Cannabinoids | 191 (1.32) | 62 (0.04) | 30.81 (23.13-41.03) |
|  | Modafinil | 1,067 (7.36) | 33 (0.02) | 323.33 (228.67-457.19) |
|  |  |  |  |  |
| **R: Respiratory system** | |  |  |  |
|  | Cetirizine | 323 (2.23) | 2,524 (1.74) | 1.28 (1.14-1.44) |
|  | Promethazine | 183 (1.26) | 1,314 (0.91) | 1.39 (1.19-1.63) |
|  | Diphenhydramine | 25 (0.17) | 90 (0.06) | 2.78 (1.78-4.33) |
|  | Acetylcysteine | 84 (0.58) | 296 (0.20) | 2.84 (2.23-3.62) |
|  | Xylometazoline | 39 (0.27) | 135 (0.09) | 2.89 (2.02-4.13) |
|  | Dichlorobenzyl alcohol | 19 (0.13) | 62 (0.04) | 3.06 (1.83-5.12) |
|  |  |  |  |  |
| **S: Sensory organs** | |  |  |  |
|  | Chloramphenicol | 787 (5.43) | 6,494 (4.48) | 1.21 (1.13-1.3) |
|  | Dexamethasone | 164 (1.13) | 980 (0.68) | 1.67 (1.42-1.97) |
|  | Artificial tears and other indifferent preparations | 258 (1.78) | 1,174 (0.81) | 2.2 (1.92-2.51) |
|  |  |  |  |  |

**Table S1**: Overview of differences in medication use at specific drug level (ATC level 5) between individuals with MS (n = 14,491) and controls (n = 144,910). Only statistically significant associations after adjustment for multiple testing with Bonferroni correction are listed.

Abbreviations: ATC: Anatomical Therapeutic Chemical classification system, CI: Confidence Interval, MS: Multiple sclerosis.

| **Medication group** | **ATC code, level 2** | **Users among females with MS,**  **n (%)** | **Users among females in the control group,**  **n (%)** | **Relative risk (95% CI)** |
| --- | --- | --- | --- | --- |
|  |  |  |  |  |
| **Increased use among females with MS** |  |  |  |  |
| Anesthetica | N01 | 48 (0.48) | 194 (0.20) | 2.47 (1.80-3.39) |
| Analgetica | N02 | 5,440 (54.70) | 36,169 (36.37) | 1.50 (1.46-1.55) |
| Antiepileptic drugs | N03 | 653 (6.57) | 2,385 (2.40) | 2.74 (2.51-2.99) |
| Antiparkinsonian drugs | N04 | 234 (2.35) | 1,077 (1.08) | 2.17 (1.89-2.50) |
| Psychoepileptic drugs | N05 | 1,552 (15.61) | 9,448 (9.50) | 1.64 (1.56-1.73) |
| Psychoanaleptics | N06 | 2,787 (28.02) | 13,884 (13.96) | 2.01 (1.93-2.09) |
| Hypophysis and hypothalamic hormones | H01 | 76 (0.76) | 359 (0.36) | 2.12 (1.65-2.71) |
| Genital hormones | G03 | 2,265 (22.78) | 18,000 (18.10) | 1.26 (1.20-1.31) |
| Corticosteroids for systemic use | H02 | 434 (4.36) | 3,418 (3.44) | 1.27 (1.15-1.40) |
| Agents for oral diseases | A01 | 490 (4.93) | 2,468 (2.48) | 1.99 (1.80-2.19) |
| Antihistamines for systemic use | R06 | 990 (9.95) | 8,667 (8,71) | 1.14 (1.07-1.22) |
| Antithrombotic agents | B01 | 919 (9.24) | 7,546 (7.59) | 1.22 (1.14-1.30) |
| Anemia medications | B03 | 723 (7.27) | 5,074 (5.10) | 1.42 (1.32-1.54) |
| Diuretics | C03 | 1,163 (11.69) | 8,671 (8.72) | 1.34 (1.26-1.43) |
| Renin-angiotensin system agents | C09 | 1,898 (19.08) | 16,945 (17.04) | 1.12 (1.07-1.17) |
| Medications for acid-related conditions | A02 | 1,956 (19.67) | 15,358 (15.44) | 1.27 (1.22-1.33) |
| Agents for functional gastrointestinal disorders | A03 | 258 (2.59) | 1,520 (1.53) | 1.70 (1.49-1.94) |
| Antiemetics | A04 | 130 (1.31) | 729 (0.73) | 1.78 (1.48-2.15) |
| Agents for constipation | A06 | 1,206 (12.13) | 2,730 (2.75) | 4.42 (4.13-4.73) |
| Agents for diarrhea and intestinal inflammation/infection | A07 | 311 (3.13) | 2,104 (2.12) | 1.48 (1.31-1.67) |
| Urologicals | G04 | 1,646 (16.55) | 964 (0.97) | 17.07 (15.77-18.49) |
| Antiinflammatory and antirheumatic agents, non-steroids | M01 | 2,193 (22.05) | 18,348 (18.45) | 1.20 (1.14-1.25) |
| Muscle relaxants | M03 | 1,930 (19.41) | 2,860 (2.88) | 6.75 (6.37-7.15) |
| Agents for the treatment of bone diseases | M05 | 683 (6.87) | 3,094 (3.11) | 2.21 (2.03-2.40) |
| Systemic antibacterial agents | J01 | 3,906 (39.28) | 27,510 (27.66) | 1.42 (1.37-1.47) |
| Systemic antifungal agents | J02 | 377 (3.79) | 2,940 (2.96) | 1.28 (1.15-1.43) |
| Systemic antiviral agents | J05 | 658 (6.62) | 4,260 (4.28) | 1.54 (1.42-1.68) |
| Vaccinations | J07 | 266 (2.67) | 1,339 (1.35) | 1.99 (1.74-2.27) |
|  |  |  |  |  |
|  |  |  |  |  |
| **Decreased use among females with MS** |  |  |  |  |
|  |  |  |  |  |
| Allergen extracts for desensitization | V01 | 7 (0.07) | 280 (0.28) | 0.25 (0.12-0.53) |
| Antidiabetic medications | A10 | 865 (8.70) | 10,100 (10.16) | 0.86 (0.80-0.92) |
|  |  |  |  |  |

**Table S2**: Medication groups with increased and decreased use among females with MS (n = 9,945) compared to females in the control group (n = 99,450). Only associations significant after adjustment for multiple testing with Bonferroni correction are listed. Topical agents are not included in the overview. Abbreviations: ATC: Anatomical Therapeutic Chemical classification system, CI: Confidence Interval, MS: Multiple sclerosis.

| **Medication group** | **ATC code, level 2** | **Users among males with MS,**  **n (%)** | **Users among males in the control group,**  **n (%)** | **Relative risk (95% CI)** |
| --- | --- | --- | --- | --- |
|  |  |  |  |  |
| **Increased use among males with MS** |  |  |  |  |
| Anesthetica | N01 | 18 (0.40) | 42 (0.09) | 4.29 (2.47-7.44) |
| Analgetica | N02 | 2,017 (44.37) | 11,648 (25.62) | 1.73 (1.65-1.82) |
| Antiepileptic drugs | N03 | 296 (6.51) | 869 (1.91) | 3.41 (2.99-3.89) |
| Antiparkinsonian drugs | N04 | 119 (2.62) | 403 (0.89) | 2.95 (2.41-3.62) |
| Psychoepileptic drugs | N05 | 529 (11.64) | 3,071 (6.76) | 1.72 (1.57-1.89) |
| Psychoanaleptics | N06 | 1,022 (22.48) | 4,081 (8.98) | 2.50 (2.34-2.68) |
| Hypophysis and hypothalamic hormones | H01 | 28 (0.62) | 29 (0.06) | 9.66 (5.74-16.23) |
| Corticosteroids for systemic use | H02 | 181 (3.98) | 1,276 (2.81) | 1.42 (1.21-1.66) |
| Agents for oral diseases | A01 | 205 (4.51) | 769 (1.69) | 2.67 (2.29-3.11) |
| Anemia medications | B03 | 270 (5.94) | 1,627 (3.58) | 1.66 (1.46-1.89) |
| Diuretics | C03 | 494 (10.87) | 3,686 (8.11) | 1.34 (1.22-1.47) |
| Vasoprotectives | C05 | 170 (3.74) | 1,245 (2.74) | 1.37 (1.16-1.60) |
| Medications for acid-related conditions | A02 | 808 (17.77) | 6,177 (13.59) | 1.31 (1.22-1.41) |
| Agents for functional gastrointestinal disorders | A03 | 65 (1.43) | 278 (0.61) | 2.34 (1.78-3.06) |
| Antiemetics | A04 | 49 (1.08) | 125 (0.27) | 3.92 (2.82-5.45) |
| Agents for constipation | A06 | 573 (12.60) | 1,127 (2.48) | 5.08 (4.60-5.62) |
| Agents for diarrhea and intestinal inflammation/infection | A07 | 92 (2.02) | 620 (1.36) | 1.48 (1.19-1.85) |
| Pancreatic hormones | H04 | 8 (0.18) | 18 (0.04) | 4.44 (1.93-10.22) |
| Urologicals | G04 | 1,373 (30.20) | 5,081 (11.18) | 2.70 (2.55-2.87) |
| Antiinflammatory and antirheumatic agents, non-steroids | M01 | 801 (17.62) | 6,819 (15.00) | 1.17 (1.09-1.26) |
| Muscle relaxants | M03 | 934 (20.55) | 771 (1.70) | 12.11 (11.01-13.33) |
| Agents for the treatment of bone diseases | M05 | 131 (2.88) | 341 (0.75) | 3.84 (3.14-4.70) |
| Systemic antibacterial agents | J01 | 1,401 (30.82) | 8.688 (19.11) | 1.61 (1.52-1.71) |
| Systemic antifungal agents | J02 | 49 (1.08) | 272 (0.60) | 1.80 (1.33-2.44) |
| Systemic antiviral agents | J05 | 145 (3.19) | 905 (1.99) | 1.60 (1.34-1.91) |
| Vaccinations | J07 | 89 (1.96) | 461 (1.01) | 1.93 (1.54-2.42) |
|  |  |  |  |  |
|  |  |  |  |  |
| **Decreased use among males with MS** |  |  |  |  |
|  |  |  |  |  |
| Arthritis urica medications | M04 | 61 (1.34) | 1,371 (3.02) | 0.44 (0.34-0.58) |
|  |  |  |  |  |

**Table S3**: Medication groups with increased and decreased use among males with MS (n = 4,546) compared to males in the control group (n = 45,460). Only associations significant after adjustment for multiple testing with Bonferroni correction are listed. Topical agents are not included in the overview. Abbreviations: ATC: Anatomical Therapeutic Chemical classification system, CI: Confidence Interval, MS: Multiple sclerosis.
